# Obstacle to the Final Removal of the Canula after Tracheotomy in Children

**Published:** 1879-11

**Authors:** 


OBSTACLE TO THE FINAL REMOVAL OF THE CANULA AFTER TRACHEOTOMY IN CHILDREN.

M. Carrie calls attention to an obstacle in the removal of the canula after tracheotomy in children, which has not formerly been described. A tracheotomized child was seized with a fit of suffocation, just as the physician was attempting the permanent removal of the canula. He perceived in the depths of the tracheal wound a reddish prominence in the interior of the trachea, which was taken for fleshy vegetation of the posterior wall. The child died in a fit of suffocation. Prof. Guyon recognized upon the post mortem specimen sent him, that the projection regarded during life as vegetation was formed by the posterior wall of the trachea itself, which was folded longitudinally in its entire thickness. This folding was itself due to the approximation of the posterior extremities of the tracheal rings, separated anteriorly for the introduction of the canula. M. Carrie after experiments concluded that this particular variety of constriction, which hitherto has not been pointed out, ought to be, nevertheless, rather frequent among children. It affeets chiefly the first three rings of the trachea. The projection which results produces a tracheal constriction that may persist and prove an obstacle to the permanent removal of the canula.—*Arch. Gen. de Med., August, 1879.*

